# Contribution of radioactive particles to the post-explosion exposure of atomic bomb survivors implied from their stable chromosome aberration rates

**DOI:** 10.3389/fpubh.2024.1335097

**Published:** 2024-01-17

**Authors:** Megu Ohtaki, Keiko Otani, Hiroshi Yasuda

**Affiliations:** ^1^Emeritus, The Center for Peace, Hiroshima University, Hiroshima, Japan; ^2^The Center for Peace, Hiroshima University, Hiroshima, Japan; ^3^Department of Radiation Biophysics, Research Institute for Radiation Biology and Medicine, Hiroshima University, Hiroshima, Japan

**Keywords:** atomic bomb survivors, chromosome aberration, dosimetry, neutron activation, radioactive particles, residual radiation exposure

## Abstract

Even today when nearly 80 years have passed after the atomic bomb (A-bomb) was dropped, there are still debates about the exact doses received by the A-bomb survivors. While initial airborne kerma radiation (or energy spectrum of emitted radiation) can be measured with sufficient accuracy to assess the radiation dose to A-bomb survivors, it is not easy to accurately assess the neutron dose including appropriate weighting of neutron absorbed dose. Particularly, possible post-explosion exposure due to the radioactive particles generated through neutron activation have been almost neglected so far, mainly because of a large uncertainty associated to the behavior of those particles. However, it has been supposed that contribution of such non-initial radiation exposure from the neutron-induced radioactive particles could be significant, according to the findings that the stable chromosomal aberration rates which indicate average whole-body radiation doses were found to be more than 30% higher for those exposed indoors than for those outdoors even at the same initial dose estimated for the Life Span Study. In this Mini Review article, the authors explain that such apparently controversial observations can be reasonably explained by assuming a higher production rate of neutron-induced radioactive particles in the indoor environment near the hypocenter.

## Introduction

The studies on health effects of the atomic bomb (hereafter abbreviated as “A-bomb”) survivors of Hiroshima and Nagasaki are regarded as invaluable epidemiological findings when discussing the health effects of radiation. This is partially because they are based on direct observation of large numbers of people (tens of thousands) and on long-term, reliable observations. In particular, the Life Span Study (LSS) conducted by the ABCC which was reorganized to the Radiation Effects Research Foundation (RERF) is a long, large-scale cohort study since 1950, five years after the atomic bombings. LSS has continued to accumulate the information on the radiation exposure and health status of A-bomb survivors, the so-called “Hibakusha” to assess their radiation doses and radiological effects. These precious databases and outcomes of LSS have greatly contributed to expanding and deepening the knowledge on health risks of radiation exposure over a long time (1–6).

However, even today when nearly 80 years have passed since the A-bombs were dropped at Hiroshima and Nagasaki, there are still debates about the exact doses received by the A-bomb survivors. In assessing the radiation doses of A-bomb survivors, initial free-in-air kerma rays (or energy spectra of emitted radiation) – assuming an unshielded environment due to nuclear testing – can be determined with sufficient accuracy, but the details of the environment at the time of the exposure of individual survivors exposed in urban areas are uncertain, and accurate assessment of the actual radiation doses is often difficult (7). In such cases, the biological dose estimates based on stable chromosome aberrations (sCA) such as translocation, inversion and deletion in lymphocyte cells obtained from the peripheral blood of A-bomb survivors can be an accurate indicator of the average radiation dose throughout the body, as they reflect the amount of injury rather than merely the physical dose (8). The authors point out here that those controversial observations on sCA among the A-bomb survivors in Hiroshima and Nagasaki imply significant contributions of radioactive particles to their post-explosion doses.

### Recent studies on chromosome aberrations of A-bomb survivors

In 2001, Kodama et al. (9) reported a clear dose–response relationship between stable chromosome aberrations in lymphocytes and the Dosimetry System 1986 (DS86). According to their paper, the subjects were a subgroup of the LSS (*n* = 1980 in Hiroshima, 1,062 in Nagasaki, and 3,042 in total) who were unshielded or shielded by wooden structures such as Japanese houses and had a detailed shielding history. Analysis of the relationship between the rate of sCA frequency and the initial dose showed a nearly linear relationship in both Hiroshima and Nagasaki. The rate of increase with dose in Hiroshima (6.6%/Sv) was about twice that in Nagasaki (3.7%/Sv), and the frequency of chromosomally aberrant cells was more than 20% higher in the indoor exposure than in the outdoor exposure for the same weighted marrow dose due to DS86. Such a low risk coefficient for Nagasaki factory workers has suggested a possible overestimation of their doses based on DS86 by about 60%.

In 2023, Sposto et al. (10) published the results of a reanalysis of the relationship between A-bomb radiation exposure and sCA frequency by FISH in 1,868 A-bomb survivors using the latest dosimetry system (DS02R1) (6). In their paper, background sCA rates and factors that may influence the shape and magnitude of the dose response were investigated. Based on the Giemsa staining method, the relationship between radiation dose and sCA rate was significant (*p* < 0. 0001), showing a linear-quadratic relationship at low doses and not persisting at high doses, which is consistent with the result of analysis of Kodama et al. using DS86 dose estimates (9); i.e., the effects of age at exposure and type of radiation shielding were significant. In contrast, the differences in the Hiroshima and Nagasaki dose responses were less pronounced (*p* = 0.026), and urban effects were not evident at doses below 1.25 Gy. Background sCA rates increased with age at examination (*p* < 0.0001), but gender, city, and smoking were not significantly associated with background rates.

**Figure 1:**
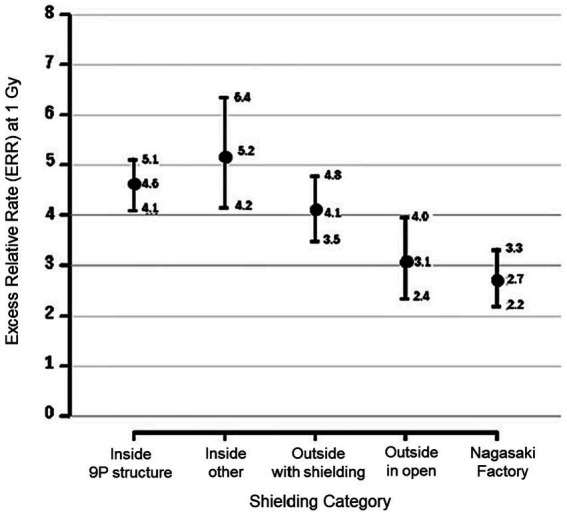
Excess relative rate (ERR) of stable chromosome aberrations (sCA) with 95%CI corresponding the aberration rate computed from the background rate in Hiroshima males aged 70 at exam at 1 Gy exposure, categorized by five shielding conditions. Reproduced and used with permission from Figure 5B of Sposto et al. (9) (© 2023 Radiation Research Society).

The most important findings of the study by Sposto et al. (10) are that the sCA frequency is 30–50% higher for survivors exposed indoors than for those exposed outdoors, and that the relative risk for factory workers in Nagasaki is very low despite the same initial dose as shown in Figure 1. These findings are essentially the same as those of Kodama et al. (10) obtained by using the previous system (DS86), and the difference in sCA dose response between indoor and outdoor conditions has suggested that the latest dosimetry system (6) still has the same issues regarding the accuracy of dosimetry.

### Explaining the paradoxical observations

As mentioned in the previous section, we have seen puzzling phenomena such as the higher incidence of sCA among those exposed indoors than among those exposed outdoors even at the same dose. This problem cannot be explained without assuming that their doses may be inaccurately given. Accordingly, in this section, the authors try to explain the reason of this controversial observations, focusing on the distance from the hypocenter to the point of exposure.

Suppose that Hibakusha A and Hibakusha B were exposed to the A-bomb radiation near the hypocenter in Hiroshima (or Nagasaki) and that Hibakusha A was exposed indoors while Hibakusha B was outdoors at the same external (DS02) dose. DS02 considered the shielding effect of the houses, so without shielding, Hibakusha A would have been exposed to a higher initial radiation dose than Hibakusha B. This means that Hibakusha A was exposed closer to the hypocenter than Hibakusha B at a higher probability. According to both DS86 and DS02, not only gamma rays but also neutrons were considered to have contributed to the exposure in the vicinity of the hypocenter at the time of the explosion.

**Figure 2:**
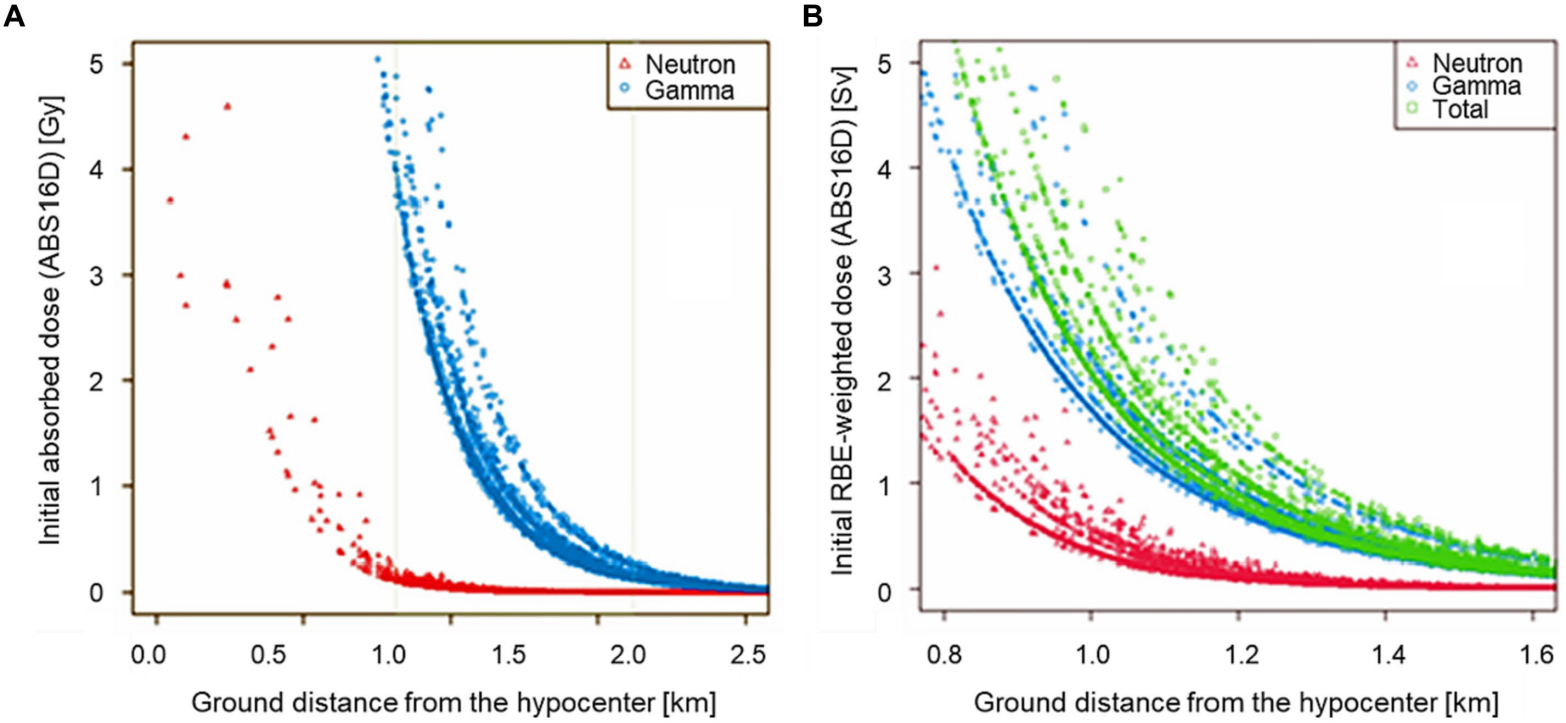
Scatter plots of the initial radiation doses of A-bomb survivors estimated with ABS16D (DS02-compliant dosimetry system managed by Hiroshima University) as a function of ground distance in kilometers from the hypocenter. The left panel shows the initial absorbed doses from 0 to 2.6 km in distance **(A)** and the right panel the RBE-weighted doses from 0.8 to 1.6 km where the neutron RBE value was assumed to be 10 **(B)**.

Figure 2A shows a scatter plot of the absorbed doses from initial exposure due to the Hiroshima A-bomb explosion as a function of the distance from the hypocenter, based on the recently established dataset (ABS16D) of Hiroshima Atomic Bomb Survivor Cohort Database (ABS) which is a DS02 compliant dosimetry system maintained at the Hiroshima University. The absorbed doses were converted to equivalent doses by the weighting of neutron absorbed dose with the RBE value of 10, as shown in Figure 2B. The graph shows that the dose due to neutrons could be significant only within 1.2 km from the hypocenter. In other words, the contribution of neutron dose could not be significant at distant places (> 1.2 km), even though a higher RBE value was employed for neutrons. Note that these ABS data presented in Figure 2 have been shared with many researchers, and part of them were published in some peer-reviewed articles (11–13).

The RBE value of 10 has constantly used in LSS by RERF, i.e., their dosimetry systems (DS86 and DS02). Even though radiobiological studies have indicated that the neutron RBE value could be significantly larger at low doses, the current value (=10) has been supposed to be reasonable for the A-bomb survivors who received relatively high doses. Cullings et al. stated that RERF had taken this RBE value in the range of total dose most relevant for linear risk estimation, namely about 1 Gy (14). They also mentioned that γ-ray risk estimation from the LSS data was hardly affected by any choice of neutron RBE value, as inferred from the dose distributions seen in Figure 2.

Based on these thoughts, the authors point out that the controversial findings on the chromosome aberrations among the A-bomb survivors (Figure 1) are attributable to contribution of radioactive particles containing neutron-activated metal elements (^27^Al, ^55^Mn, ^23^Na, etc.) in the ground surface and building materials. It is probable that a highly radioactive contaminated area was formed around the hypocenter immediately after the A-bomb explosion, and a large amount of those neutron-induced radioactive particles was stirred up and diffused in air to surrounding areas owing to the bomb blast. It should be noteworthy that there are many reports from Hibakusha in both Hiroshima and Nagasaki who were near the hypocenter observed that the sunlight was blocked by dust and darkness soon after the explosion (15). Under such circumstances, it is probable that they were exposed to radiations from various neutron-induced radionuclides in the broken building materials and ground surface soils (16). As the neutron activations can occur almost simultaneously with the A-bomb explosion, the people within a few kilometers from the hypocenter are presumed to have begun inhaled those hot particles including various short-lived radionuclides, regardless of their pre-explosion shielding conditions.

While many of the Hibakusha who stayed in outdoors and were not fatally injured immediately left the hypocenter area for safer places, it is presumed that the Hibakusha who stayed indoors near the hypocenter continued to be exposed to an environment filled with radioactive aerosols for a longer period. The fact that chromosome exams were conducted more than 20 years after the bombings means that the Hibakusha who received the exams had survived for at least 20 years. In other words, only those Hibakusha who were able to evacuate the area on the day of the bombings without sustaining fatal injuries would be eligible for chromosome exams. It is likely that the Hibakusha who was closer to the hypocenter were exposed to more radioactive particles for a relatively long time through the evacuation activities. That is, it is considered that Hibakusha A would have had a higher probability of being exposed to more non-initial radiation than Hibakusha B. Even though the time delay were several tens of minutes, it could lead to significant differences in the radiation dose in comparison to the length of half-lives of the major neutron-induced radionuclides such as ^28^Al (half-life is 2.2 min), ^56^Mn (half-life is 2.6 h), and ^24^Na (half-life is 15 h). As a result, it is considered that Hibakusha A received significantly higher radiation dose than Hibakusha B under the condition that the initial external (DS02) doses of both groups were the same.

Considering the potentially significant contribution of non-initial radiation exposure from the radioactive particles, the authors think that it cannot be justified to assume different neutron spectra or a higher value of neutron RBE to explain the incidence/mortality data of A-bomb survivors’ cohort in Hiroshima and Nagasaki who were assessed with the initial external radiation doses only. On the other hand, by taking into account the large contribution of radioactive particles, we can explain consistently the results of chromosome aberrations and also the cohort data of the A-bomb survivors without consideration of changing the current neutron spectra or neutron RBE value.

In more detail, the observations that the relative excess rates of sCA were higher than 30% for the A-bomb survivors exposed indoors than for those exposed outdoors near the hypocenter even when the initial external doses were the same (Figure 1) can be explained as follows. The fact that the initial dose for those who were indoors was 1 Gy means that they would have been exposed to stronger initial radiation outdoors around their homes, especially if they were closer to the hypocenter within 1.2 km from the hypocenter in Hiroshima (Figure 2), where they would have been exposed to neutrons produced in the A-bomb explosion and contaminated by high levels of radioactivity through activation. On the other hand, A-bomb survivors with an initial dose of 1 Gy from outdoor exposure have a high probability of being at a longer exposure distance at 1.2 km or more from the hypocenter in Hiroshima, and are unlikely to have been affected by strong neutron activation. Therefore, the probability of the total dose including exposure other than the initial dose is higher for those with an initial dose of 1 Gy.

As for the quite low risk Nagasaki factory workers shown in Figure 1, it is noteworthy that at the time of the bombing, in the suburbs of Nagasaki, a southerly sea breeze was blowing in the middle of the day on a fine day in mid-summer, and many factories located near the coast were located upwind of the hypocenter (in the southwest direction from the hypocenter). Therefore, it is assumed that Nagasaki factory workers were able to avoid exposure to radioactive particulates. That is, factory workers in Nagasaki may have received little additional radiation exposure from noninitial radiation from the A-bomb explosion. Such strange evidence, i.e., the low risk of the factory workers in Nagasaki and the difference in effects between indoor/outdoor exposure situations can be consistently explained, if the effects of additional exposure from radioactive particles generated in the vicinity of the hypocenter are taken into account.

It has been also pointed out that the above-mentioned controversy can attribute to underestimation of current neutron doses. According to the fact that the neutron spectra used in dose calculation for the A-bomb survivors in LSS agreed well with the experimental results (15), another possible explanation is that the current fixed value (=10) of neutron RBE may be significantly smaller than the real value. Regarding this possibility, some recent studies suggested that the neutron RBE to be applied for A-bomb survivors could be much higher (12, 17, 18). Also, some *in-vitro* studies on chromosome aberrations in human lymphocytes support such high values of neutron RBE (19–22). However, such high values of neutron RBE were obtained at low dose levels concerned for the aim of radiological protection (23) and the neutron RBE tends to be significantly smaller at higher dose around the order of Gy (20) comparable to that received by the A-bomb survivors near the hypocenter. Regarding this issue, Cullings et al. (14) pointed out that application of the pure neutron field RBE to the mixed-field A-bomb radiation was a questionable approach which would result in overestimation of the actual neutron RBE for moderate total dose levels of 1 Gy by a factor of more than four. Actually, in the criticality accident in Tokaimura, Japan, the RBE value of 1.7 was reasonably employed for the high-dose neutrons generated in the fission reactions (24, 25). Accordingly, application of a high RBE value greater than 10 cannot be justified for the A-bomb survivors who received the high-dose exposures that induced the deterministic effects (tissue reactions).

### Contribution of radioactive particles to health risk

We have not formed yet a consensus about the health effects of the indirect exposure from residual radiation emitted from the radioactive materials that were caused mainly by the A-bomb neutrons. While the inability to gain a direct estimation of the radiation dose has led to an assumption that the dose of residual radiation would be several tens of mGy at most (26), some clinicians and researchers have pointed out that the onset of acute radiation injury (hereinafter referred to as “acute symptom”) and the solid cancer incidence/mortality among A-bomb survivors could not be explained only by the external dose immediately after the bombings (27). O-ho (28) found that the incidence of each acute symptom decreased with increasing distance from the hypocenter when there was no access to the hypocenter (within 1 km of the hypocenter) within 3 months after the bombing, and that the incidence of each acute symptom was higher when there was access to the hypocenter within 3 months than when there was no access; they also found that even those who were not in Hiroshima City at the time of the bombing developed acute symptoms and the prevalence of acute symptoms was not necessarily directly correlated with the distance from the hypocenter, which suggested that the effects of residual radiation received in the hypocenter area were underestimated. According to these findings, Sawada (29) proposed a hypothesis on the mechanism of the effects of residual radiation exposure on the acute symptoms of A-bomb survivors.

Some independent researches using ABS conducted independently from LSS indicated that solid cancer mortality risk among A-bomb survivors was higher in the west of the hypocenter, i.e., a non-circular symmetry distribution (30–32). In addition, Otani et al. (33) have reported that the risks of solid cancer mortality of the people who entered the area affected by the atomic bombing in Hiroshima City on August 6 and 7, 1945 but received no initial radiation exposures were estimated to exceed by 18 and 7%, respectively, when compared with those who entered the same area few days later (August 9, 1945). Kamada et al. (34) have also detected that the risk of leukemia among entrants to the city for the period 1970–1990 was 3.7 times higher for both males and females when the date of entry was August 6, 1945, compared to other Japanese nationals during the same period.

It is obvious that explosion of A-bomb can generate various radioactive fission and particle activated products through neutron activation reactions as well as radioactive fallout (35–37), and the exposure to the radioactive particles generated by the nuclear explosion can cause chromosome aberrations of survivors. As a study relevant to this issue, Tanaka et al. (38) examined sCA in peripheral blood lymphocytes of 17 crew members of eight fishing vessels and two crew members of one cargo ship in detail by the G banding method 60 years after the nuclear tests conducted by United States at the “Bravo” hypocenters on Bikini Atoll and Eniwetok Atoll in the Marshall Islands; those crew of tuna fishing boats and cargo ships were operating approximately 150–1,200 km from the test sites at the bombing and received exposures to the radioactive fallout. Compared to 9 age-matched controls, the percentage of stable-type abnormalities was 3.35% in the exposed group, which was significantly higher than that (2.45%) in the control group.

Although there are not a few epidemiological observations of the above-mentioned health effects of radioactive particles, there are few reports from animal experiments to explain these effects. As an only exceptional study, Hoshi and his team showed that there are notable, specific effects that have not been previously found for exposure to radioactive microparticles (39). They suggested that the health effects of exposure to radioactive particles can be 20 times greater than those of external exposure at the same absorbed dose, though the mechanism behind these results is yet to be investigated in future studies.

## Conclusion

In this Mini Review article, the authors explained that the A-bomb detonation could cause significant additional exposure from the radioactive particles generated through neutron activation, particularly near the hypocenter. This explanation can solve the controversial issue regarding the observed difference of chromosome aberrations between the indoor and outdoor A-bomb survivors more convincingly than other approaches such as reconsideration of neutron energy spectra or assumption of an unreasonably high value of neutron RBE. The authors believe that such a convincing explanation would positively change the attitudes of health care workers of whom many feel uncomfortable and unprepared for dealing with radioactively contaminated patients in a future nuclear incident (38).

It is expected that further quantitative studies on the relevant issues (types and amounts of elements contained in the building materials when the A-bomb was dropped, levels of neutron activation of those elements due to the A-bomb neutrons, resultant doses of both external and internal exposures, etc.) will improve the reliability of impact assessment of a possible nuclear detonation, criticality accident and terrorist attack using fissionable materials, as part of nuclear emergency preparedness and responses.

## Author contributions

MO: Conceptualization, Data curation, Formal analysis, Funding acquisition, Investigation, Methodology, Software, Writing – original draft. KO: Data curation, Formal analysis, Methodology, Validation, Writing – review & editing. HY: Funding acquisition, Investigation, Supervision, Validation, Visualization, Writing – review & editing.
